# Sleep bruxism in children, from evidence to the clinic. A systematic review

**DOI:** 10.3389/froh.2023.1166091

**Published:** 2023-05-11

**Authors:** Claudia Restrepo-Serna, Efraim Winocur

**Affiliations:** ^1^ CES-LPH Research Group, Universidad CES, Medellin, Colombia; ^2^ Department of Oral Rehabilitation, the Maurice and Gabriela Goldschleger, School of Dental Medicine, Sackler Faculty of Medicine, Tel Aviv University, Tel Aviv, Israel

**Keywords:** sleep bruxism, children, anxiety, sugar, screen time, obstructive sleep apnea, sleep architecture

## Abstract

**Objectives:**

The present paper aims to systematically review the literature published from 2015 to 2023 on bruxism in children with the aim to compilate the best available evidence.

**Materials and Methods:**

A systematic search in the National Library of Medicine's PubMed, Medline (EBSCO), SCOPUS, and Google Scholar databases was performed to identify all studies on humans assessing genetic, biopsychosocial, and sleep factors assessed with any different approach for sleep bruxism (SB) in children and its interventions. The selected articles were assessed independently by the two authors according to a structured reading of the article's format (PICO). The quality of the articles was evaluated using Quality Assessments Tool for Experimental Bruxism Studies (Qu-ATEBS) and the JBI critical appraisal tools.

**Results:**

A total of 16 articles were included for discussion in the review and grouped into questionnaire/parental-report (*n* = 7), SB assessment through parental report of SB and clinical examination (*n* = 4), and instrumental assessment (*n* = 5) studies. The total quality scores evaluated with STROBE and Qu-ATEBS were high for all included papers. However, in general, there was no control of bias strategies and there was no control group in the intervention studies.

**Conclusions:**

Investigations based on self-report, clinical, and instrumental bruxism assessment showed a positive association with genetics, quality of life aspects (school and emotional functions and overuse of screen-time), mother anxiety and family conformation, diet, alteration in sleep behaviors and architecture, and sleep breathing disorders. Additionally, the literature presents options to increase airway patency and, thus, reduce the occurrence of SB. Tooth wear was not found to be a major sign of SB in children. However, methods of SB assessment are heterogeneous and hamper a reliable comparison of the results.

## Introduction

Sleep bruxism (SB) is a repetitive jaw-muscle activity characterized by clenching or grinding of the teeth and/or by bracing or thrusting of the mandible during sleep (1). The concept of bruxism shifted from a disorder to a motor activity that may even have potential protective relevance (2, 3).

The etiology of SB is multifactorial (4) and includes biological, psychosocial, and lifestyle factors. According to gene analysis studies (5, 6) and studies among individuals of the same family (7), SB is explained by both environmental and genetic factors (8, 9). Moreover, an imbalance in certain neurotransmitters in the central nervous system (e.g., dopamine and serotonin) play a role in the genesis of masticatory muscle activity (MMA) and SB (10). This imbalance is related to lifestyle factors, like consumption of added sugar and screen-time overuse (11), and psychosocial factors, such as anxiety, depression, and stress, which have also been demonstrated to increase risk for SB (12, 13). Additionally, health issues like breathing disorders (14) and Gastroesophageal Reflux Disease (GERD) (15) have also been suggested as predictors of SB.

Studies that follow cohorts to assess SB are scarce, which hampers the possibility of establishing a cause-consequence relationship. SB could be a transitory activity related to positive consequences in most of the cases (such as reducing cortisol levels during positive stress situations), but in some cases is the symptom of situations that can even compromise the patient's life (obstructive sleep apnea). In this last point, following subjects over time without undergoing intervention for the underlying condition is not viable due to ethical concerns.

Variations in the conception and assessment of SB, population characteristics, and research methodologies among different studies might partially contribute to the inconsistent results. Due to these reasons, clinicians lack enough tools to update and apply an adequate evidence-based dentistry regarding bruxism in children. Therefore, this review aimed to compile the best available evidence about bruxism in children over the last seven years and give evidence-based recommendations for pediatric healthcare givers to treat bruxism in the clinic. The question we aim to answer is, “What is the evidence that supports the assessment and intervention of sleep bruxism in children?”

## Materials and methods

This systematic review was registered in PROSPERO.

### Search strategy

On March 25 2023, a literature review was performed based on a search in PubMed, Medline (EBSCO), SCOPUS, and Google Scholar databases for articles on SB in children and its etiology, assessment, and intervention. The search was filtered to include only papers published from 2015 to 2023 in the English language. The keywords used in the search strategy for each database are included in Table 1. The article screening included two phases: title and abstracts screening, and full-text review. First, all identified titles and abstracts were independently screened by the two authors. The inclusion criteria were: (a) studies on child human subjects (age 0–18 years); (b) studies dealing with SB, evaluated by parental-report [e.g., reporting of sleep tooth-grinding sound (STG) by questionnaire or interview] and/or clinical inspection (e.g., tooth wear, Linea Alba, and/or masticatory muscle pain), and/or instrumental assessment (e.g., scoring of SB episodes based on polysomnography (PSG), polygraphy, or Electromyography (EMG)); and (c) studies having the following designs: observational studies, controlled clinical trials, or randomized controlled clinical trials. The exclusion criteria were: (a) studies on animals; (b) studies on adults; (c) publication types such as editorials, letters, legal cases, interviews, and conference abstracts; (d) studies in which the assessment of SB and/or the outcome measures were not performed with calibrated/validated instruments and/or techniques; (e) studies including children with neurodevelopmental disorders; and (f) retrospective design. Full texts of eligible studies were only obtained when both reviewers were in consensus. Lack of agreement between the reviewers was resolved by discussion between both investigators.

**Table 1:** Search terms for each database.

Database	Terms
PubMed, Medline (EBSCO), SCOPUS	(“Bruxism”[Mesh] OR “Sleep Bruxism” [Mesh] OR “Teeth Grinding Disorder”[Title/Abstract]) OR “Teeth Grinding Disorders”[Title/Abstract] OR “Sleep Bruxisms”[Title/Abstract] OR “Nocturnal Teeth Grinding Disorder”[Title/Abstract] OR “Nocturnal Bruxism”[Title/Abstract] OR “Nocturnal Bruxisms” [Title/Abstract] OR “Childhood Sleep Bruxism” [Title/Abstract] OR “Childhood Sleep Bruxisms” [Title/Abstract] OR “Sleep-Related Bruxism”[Title/Abstract] OR “Sleep-Related Bruxisms” [Title/Abstract] OR bruxism [Title/Abstract] OR “teeth grinding” [Title/Abstract] OR “Sleep bruxism” [Title/Abstract]) AND (children [Title/Abstract] OR child [Title/Abstract] OR kid [Title/Abstract] OR kids [Title/Abstract] OR “Child”[Mesh])
Google Scholar	“Sleep Bruxism Children”, “Sleep Bruxism Children Sleep Architecture”, “Sleep Bruxism Children Polysomnography”, “Sleep Bruxism Children PSG”, “Sleep Bruxism Children Electromyography”, “Sleep Bruxism Children EMG”, “Sleep Bruxism Children Stress”, “Sleep Bruxism Children Anxiety”, “Sleep Bruxism Children Sugar”, “Sleep Bruxism Children Screens”, “Sleep Bruxism Children Questionnaires”, “Sleep Bruxism Children Assessment”, “Sleep Bruxism Children Treatment”, “Sleep Bruxism Children Intervention”

### Assessment of papers

The selected articles were read and assessed independently by the two authors according to a PICO (Population-Exposure-Comparison-Outcome) structured strategy. The population (P) is described in terms of sample size, inclusion criteria, and demographic characteristics. The intervention (I) concerns information on the study design, assessment approach, and measurement of variables. The comparison (C) includes data on the control group depending on the study design (i.e., individuals without SB). The outcome (O) is reported in terms of the possible relationship between different biopsychosocial and lifestyle variables and SB in children. The main conclusion of each study was also included.

The PRISMA checklist for systematic reviews was followed.

### Risk of bias (quality assessment)

Strengthening the Reporting of Observational studies in Epidemiology (STROBE) (16) was used for the quality assessment of case-control and cross-sectional studies. STROBE score, defined as the number of the 22 STROBE items adequately reported divided by the number of applicable items, expressed as a percentage, was determined for the quality assessment of the studies. The 13 STROBE items with several questions (2–15 questions per item, online supplementary) were considered adequately reported when at least 50% of their questions had “yes” answers (after exclusion of the “not applicable” components).

Those studies dealing with intervention of sleep bruxism in children were evaluated with the Quality Assessments Tool for Experimental Bruxism Studies (Qu-ATEBS) (17). A score between 0 and 50 was considered low quality and a score between 51 and 70 was considered high quality. Only studies with a score above 50% in STROBE and above 51 in Qu-ATEBS were included in this systematic review.

## Results

**Figure 1:**
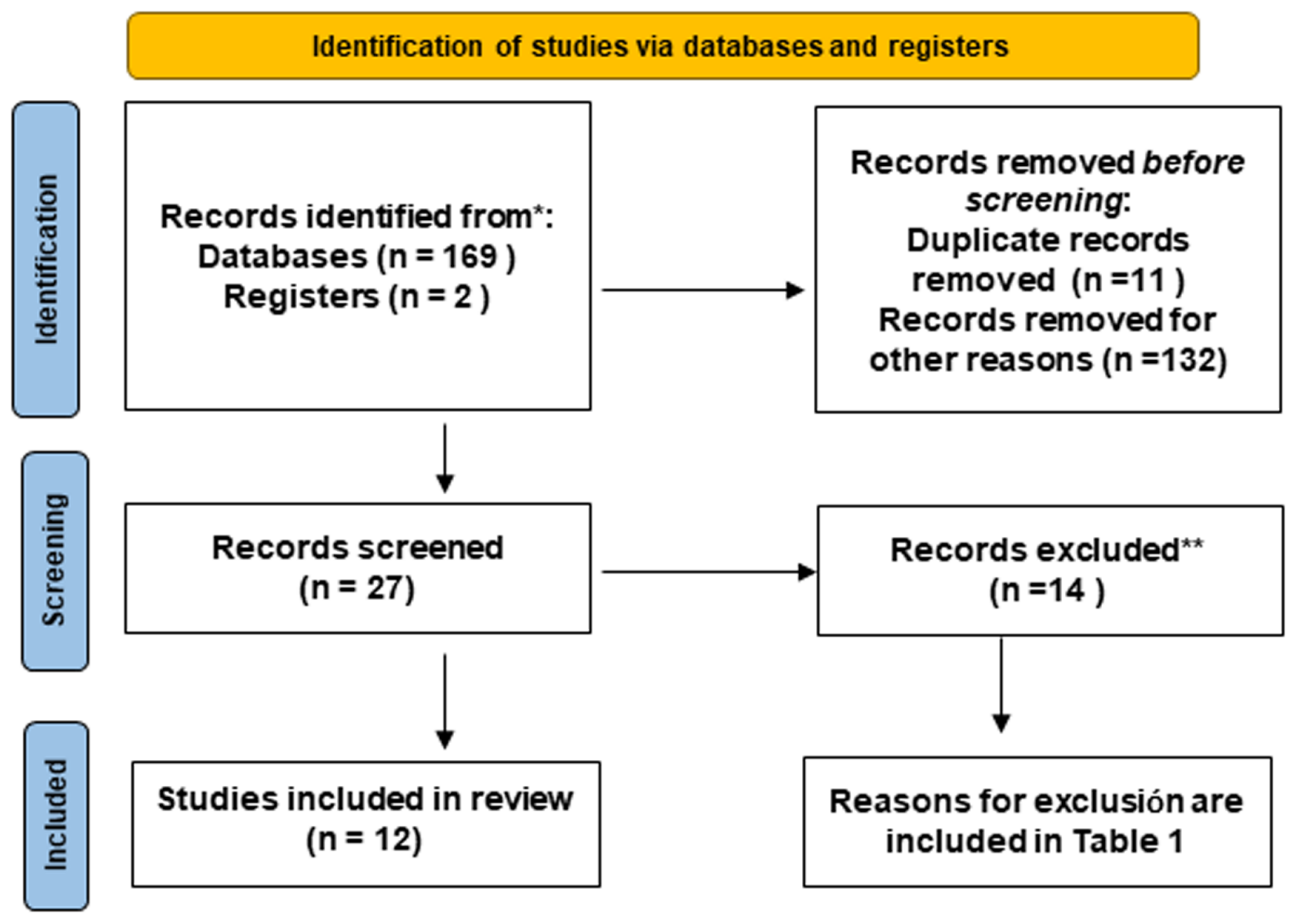
PRISMA flow diagram for selection of studies.

Title and abstract reading led to the exclusion of 143 irrelevant articles. The full text was obtained of the remaining 27 articles. Of these, 14 were excluded for not fulfilling the inclusion criteria, so that 13 articles were selected for inclusion in the review. Two more papers were added to the review by searching the reference lists of two previous papers (18, 19). Thus, a total of 16 articles were included in the review. Table 2 includes the list of articles and the reason for exclusion from the review after reading the full text. Based on the type of assessment of SB, the selected articles were then divided into three categories: SB assessment by means of parental report (*n* = 7), SB assessment through parental report of SB and clinical examination (*n* = 4), and instrumental assessment (*n* = 5). The flow diagram for the inclusion of the studies is presented in Figure 1.

**Table 2:** Studies retrieved in full text but not included in the review.

Study first author and year	Reason for exclusion
Lin, 2021 (20)	No information about the smoking-SB association itself.
Sampaio, 2018 (21)	One of the selection criteria was used as outcome measure (tooth wear)
Kobayashi, 2022 (22)	No information about SB assessment is provided.
Oliveira, 2015 (23)	No validated instruments were used to evaluate anxiety and behavior (outcome measures)
Alfano, 2018 (24)	The assessment of SB was made through parental-report and PSG, so selection criteria are not well defined.
de Alencar, 2017 (25)	Outcome measures are not clear.
Ceyhan, 2022 (26)	The rationale of the study is not well established.
Soares-Silva, 2019 (27)	The exclusion criteria included dental erosion, so many false negatives were possible.
Huynh, 2016 (28)	Subjects were selected based on a questionnaire, but subjects in the control group were selected for comparisons according to PSG data.
Bellerive, 2015 (29)	Subjects were randomized according to the type of device for palatal expansion. However, the groups were not comparable, as SB frequency was different between the intervention and control groups.
Martins, 2022 (30)	The outcome measure (attrition tooth wear) was not evaluated with a calibrated instrument.
Gomes, 2018 (31)	Tooth wear is used in the assessment of SB as part of the inclusion criteria and also as an outcome measure.
Massignan, 2019 (32)	Tooth wear is used in the assessment of SB as part of the inclusion criteria and also as an outcome measure.
Tavares-Silva, 2019 (33)	Not validated instrument is reported for the assessment of SB.

The total quality scores evaluated with STROBE for all included papers with cross-sectional, case-control, and epidemiological studies were high. In general, there was no control of bias strategies. The total quality scores evaluated with Qu-ATEBS were high for the two included papers. However, in general, there was no control of bias and there was no control group in the intervention studies.

### Summary of studies based on questionnaire/self-report of SB

Seven studies investigated the association between parental-reported SB and lifestyle, habits, psychological and stress aspects, tooth wear, and the effect of two interventions (direct and indirect) for SB in children (Table 3).

**Table 3:** PICO-like structured Reading of reviewed articles using parental-reported SB as assessment strategy.

Study First author and year	Patient (Population)	Intervention (Design)	Comparison (Control Group)	Outcome	Conclusions	Quality SCORE
Restrepo C, 2021 (34)	One-hundred fourteen 4–8 year-old Colombian children presented either “Sometimes” (2–4 nights) or “Usually” (5–7 nights) according to the parental report of STG in the CSHQ.	Design: Descriptive cross-sectional epidemiological study was performed. Measurements: Frequency of STG was assessed with the CSHQ. The Health Behavior in School-Aged Children Food-Frequency Questionnaire (HBSC-FFQ) was used to assess the frequency of consumption of added sugar, and the time children spent using screens was reported daily by parents on weekdays and the weekend for a week.	One-hundred eighty-six 4–8 year-old children whose parents answered “Never-Rarely” (0–1 night) STG in the CSHQ.	Correlations of SB in children were statistically significant with screen-time (Rho ¼ 0.8; *p* ¼ 0.002) and sugar-consumption (Rho ¼ 0.7; *p* ¼ 0.03). Associations were found between possible SB and increase-to-increase screen-time and sugar-consumption (OR > 2).	Overuse of screen-time and consumption of added sugar over twice a day are predictors of SB in children.	90%^a^
Restrepo C, 2017 (35)	SB was reported in the CSHQ as “sometimes” by the parents of 130 Colombian 6–13 year-old children and “usually” by the parents of 126.	Design: A stratified population-based observational study was performed. Measurements: The CSHQ was filled out by the parents of all participants to evaluate sleep behaviors in children with SB.	SB was reported as “rarely” by the parents of 1089 Colombian 6–13 year-old children in the CSHQ.	Most sleep patterns and daytime sleepiness were similar for children with different frequencies of parental-reported SB, while breathing sleep disorders and parasomnias increased with the frequency of parental-reported SB (Bonferroni post-hoc <0.001). The association between sleep habits and the frequency of parental-reported SB was different for each socioeconomic layer.	Sleep disorder breathing and parasomnias seem to be associated with parental-reported SB in children. The influence of socioeconomic conditions on sleep behaviors was not relevant.	95%^a^
Manfredini, 2017 (36)	SB was reported in the CSHQ as “sometimes” by the parents of 168 children and “usually/always” by the parents of 240. Children were 6–13 years-old.	Design: A stratified population-based observational study was performed. Measurements Parents filled out the Pediatric Inventory of Quality of Life (PedsQL4.0™) and were asked about their sociodemographic and socioeconomic conditions. Associations between such proxy-reported SB and QoL features were assessed.	“Never/Rarely” SB was the answer in the CSHQ of 1148 parents of 6–13 year-old children.	No significant associations were shown between parental reported SB and the total and domain PedsQL scores, except for a correlation with the School Functioning Score. As for the specific QoL items, only two variables of the Emotional Functioning Scale of the PedsQL4.0 (feeling afraid or scared and trouble sleeping) and a feature of the School Functioning Scale (forgetting things) were correlated with SB (*p* < 0.05).	Affection of school functioning (forgetting things) and emotional functioning were identified as associated factors with SB. However, the correlation coefficients were not high.	87%^a^
Yazıcıoğlu İ, 2022 (37)	Seven- to eleven-year-old children with SB (*n* = 48) were selected according to the AASM criteria: 1) Parental report of STG. 2) Absence of medical or mental disorders associated with SB (eg, sleep-related epilepsy or abnormal movements during sleep). 3) No other sleep disorders (eg, obstructive sleep apnea).	Design: Case-control study. Measurements: Anxiety was assessed in children with the Screen for Child Anxiety and Related Disorders (SCARED) and the State-Trait Anxiety Inventory (STAI) was used for the measurement of anxiety in mothers.	Forty-eight 7–11 year-old children in the control group were also compatible with the second and third AASM criteria.	In children, separation anxiety (*p* = 0.015), social anxiety (*p* = 0.011), and school fear (p = 0.005) were higher in children with SB than in controls. In mothers, state anxiety was significantly higher in those whose children presented SB (*p* < 0.001). Learning, behavioral, and anger problems, stress, snoring, and increased anxiety levels of mothers and children were statistically significant predictors of SB in children (*p* < 0.05).	Elevated anxiety levels of mother and children, learning, behavioral, and anger states, and snoring were found to be risk predictors of SB in children.	90%^a^
Garmroudinezhad Rostami, 2020 (38)	Mothers of 459 1.5- to 6-year-old children answered “often” or “always” to the question “Does your child grind his/her teeth during the night?” in the Self-Administered Questionnaire for Mother.	Design: A cross-sectional observational study, part of the Québec Longitudinal Study of Child Development. Measurements: Separation anxiety scores were measured with the Interviewer-Completed Computerized Questionnaire.	Mothers of 866 1.5- to 6-year-old children answered “never” or “sometimes” to the question “Does your child grind his/her teeth during the night?” in the Self-Administered Questionnaire for Mother.	Sleep bruxism and separation anxiety trajectories were weakly associated (X2 = 37.84, *p* < 0.001). Compared with preschoolers belonging to the Low-Persistent separation anxiety trajectory, preschoolers in the High-Increasing separation anxiety trajectory had almost double the risk of presenting sleep bruxism at age 7 (95% CI = 1.25–3.22, *p* = 0.04).	When separation anxiety issues are detected in early childhood, it would be useful to target sleep bruxism during the first year of elementary school.	85%^a^
Restrepo, 2017 (39)	Parents of 36 6–12-year-old children reported STG as “Sometimes” (2–4 nights) or “Usually” (5–7 nights) by means of the (CSHQ).	Design: Cross-sectional observational study. Measurement: Tooth Wear of 1637 teeth was evaluated using the screening module of the Tooth Wear Evaluation System (TWES). Dietary habits were investigated by means of the Health Behavior in School-Aged Children Food-Frequency Questionnaire (HBSC-FFQ).	Parents of 97 6–12-year-old children reported “Never-Rarely” (0–1 night) STG in the CSHQ.	Parental-report of STG is not associated with tooth wear in the mixed dentition. Associations were found between dietary habits and the severity of both incisal/occlusal and non-incisal, non-occlusal tooth wear (OR > 2).	There was not a strong correlation of STG with tooth wear in the mixed dentition. However, dietary habits (added sugar) showed to have effects in terms of increasing severity of tooth wear.	85%^a^
Giannasi, 2015 (40)	Fourteen children aged 4–11 years with anterior open bite, snoring, and bruxism. Parents/guardians were instructed to keep a modified sleep diary, with specific questions (yes/no responses) addressing SB.	Design: Quasi-experimental. Intervention and measurements: McNamara rapid maxillary expansion device was used by the children. The expansion protocol was ¼ turn in the morning and ¼ turn at night for a 7–10 day period, and the McNamara device was removed after 6–8 months. A questionnaire was administered before starting and after 30 days of use of the orthopedic appliance, addressing signs of sleep disorder, such as tiredness upon waking, mood, nightmares, movements during sleep, lip seal, drooling, snoring, and SB.	The same group was evaluated before and after intervention.	Significant improvements were found in tiredness upon waking (*p* = 0.002), mood (*p* = 0.008), lip seal (*p* = 0.031), drooling during sleep (*p* = 0.031), snoring (*p* = 0.001), and bruxism (*p* = 0.0062)	The use of rapid palatal expansion can be an effective treatment for snoring and thus reduce the prevalence of SB in children.	50^b^

^a^ Quality assessment with STROBE.

^b^ Quality assessment with Qu-ATEBS.

Study designs were strongly heterogeneous, based on the evaluation of SB either in non-patient children (e.g., school students or pediatric patients attending dental clinics of universities for conservative care) or in case-control groups of SB. Methodological differences were also evident from the strategy to assess SB as well as from the outcome measures established in each investigation. All the studies were based on a cross-sectional assessment, except for the two intervention studies. The authors used parental reports based on the Children's Sleep Habits Questionnaire (CSHQ) (34–36, 39) and the Self-Administered Questionnaire for Mother (38) and one used only the report included in the criteria of the American Academy of Sleep Medicine (AASM) (37).

As for the results, six papers found a statistically significant association between SB and breathing disorders and parasomnias (35), forgetting things and feeling afraid or scared (36), and overuse of screen time and added sugar (34). Demographic characteristics were found not to affect the frequency of SB in children (35, 36) and tooth wear was not found to be significantly associated with the frequency of SB in children with mixed dentition (39).

The paper dealing with palatal expansion on SB in children (40) showed reduction of parental-reported SB within the time of the study, but did not present long-term results.

### Summary of studies based on parental-report and clinical assessment

Four investigations calculated the correlation of SB evaluated by means of parental report and clinical examination (tooth wear, Linea Alba, etc.) with genetics and family function (Table 4). All studies except one (44) were based on a case-control design (41–43) with comparison groups of SB vs. non-SB in children. The authors used parental reports of SB and a comprehensive clinical assessment (41, 42) or parental-reported SB and presence of tooth wear (43, 44) to evaluate bruxism.

**Table 4:** PICO-like structured Reading of reviewed articles using parental report and clinical assessment of sleep bruxism as assessment strategy.

Study First author and year	Patient (Population)	Intervention (Design)	Comparison (Control Group)	Outcome	Conclusions	Quality SCORE
Calvano, 2020 (41)	Sixty-one Brazilian children (mean age 9.34 years) were considered bruxers when there was parental-reported tooth clenching and or STG during sleep, dental wear, dental impressions on the cheek mucosa and tongue, headaches, and orofacial pain on awakening, chewing and or opening, or tenderness and pain on the temporal and masseter muscles bilateral palpation.	Design: Case-control Measurements: Analysis of three genetic polymorphisms in the ACTN3 (rs678397, rs1671064, and rs1815739) in saliva using PCR. Associations were made between polymorphisms and SB.	Ninety children without SB (mean age 9.34 years).	There were associations between genetic polymorphisms rs678397, rs1671064, and rs1815739 and SB in the co-dominate model and in the recessive model (*p* < 0.05). Allele distribution for the polymorphisms rs678397 and rs1671064 was also related to SB (*p* < 0.05).	Associations between the genetic polymorphisms rs678397, rs1671064, and rs1815739 in ACTN3 and SB were found and may contribute to the etiology of SB in children.	80%^a^
Scariot, 2022 (42)	Sixty Brazilian children (mean age 9.34 years) were considered bruxers when there was parental-reported tooth clenching and or STG, dental wear, dental impressions on the cheek mucosa and tongue, headaches, and orofacial pain on awakening, chewing, and or opening, tenderness and pain on the temporal and masseter muscles during bilateral palpation.	Design: Case-control. Measurements: Analysis of single nucleotide polymorphisms in FKBP5, DRD2, ANKK1, and COMT with PCR in saliva. Associations were made between polymorphisms and SB.	Ninety children without SB (mean age 9.34 years).	SB was associated with DRD2 (*p* = 0.02). STG was associated with DRD2 in the additive (*p* = 0.030) and dominant (*p* = 0.008) model, while tooth clenching during sleep was associated with ANKK1 (*p* < 0.001) and with COMT in the additive (*p* = 0.001) and dominant (*p* = 0.003) models.	SNP in the DRD2 gene was associated with bruxism and its circadian phenotype, whereas SNPs in ANKK1 and COMT were found to be associated with circadian phenotypes of bruxism.	78%^a^
Drumond, 2020 (43)	One-hundred sixty 8–10-year-old children with SB were included. SB was assessed based on a combination of self-reports or parental reports of STG or clenching as well as a clinical examination for the analysis of abnormal tooth wear, according to Smith and Knight and/or muscle discomfort.	Design: A case-control study was performed. Measurements: Parents answered a questionnaire about parafunctional habits; they also answered the “Adaptability and Cohesion Evaluation Scales (FACES III)” to evaluate Family Functioning and the Lipp Stress Symptoms Inventory (LSSI) for adults to determine mother stress.	One-hundred sixty 8–10 year-old children without SB matched for sex and age.	SB was present in 67.3% of children with stress. Child's stress (Adjusted OR = 2.22; *p* = 0.013) and nail biting (Adjusted OR = 2.22; *p* = 0.001) were risk predictors of SB.	Children's Stress, mother's stress, and a history of nail biting are important signs to be considered in school children with SB.	90%^a^
Oh, 2021 (44)	Parents of 23 6–12 year-old children were determined to have SB according to question #19 of the SDSC which asks the frequency of STG and clinical examination for tooth wear. Children had to meet both criteria: Parental report of STG as occasional or always and presence of mild-severe dental wear.	Design: Cross-sectional. The FAIREST-15 clinical examination screening tool was used to evaluate breathing, posture, concentration, and anxiety. Tonsillar hypertrophy was assessed by means of the Brodsky scale and nasal obstruction based on the nasal breathing test.	Seventy-three 6–12-year-old children did not present either parental report of SB or tooth wear.	The prevalence of SB in subjects with tonsil hypertrophy, restricted tongue mobility, and nasal obstruction was 90.9% (*p* < 0.0001)	Tonsil hypertrophy, restricted tongue mobility, and nasal obstruction are associated with SB. Dentists should evaluate tonsils, tongue mobility, and nasal obstruction when assessing SB.	90%^a^

^a^ Quality assessment with STROBE.

The two studies dealing with genetics found certain polymorphisms of Dopamine and Serotonin associated with bruxism (*p* < 0.05) (41, 42). Another study concluded that nail-biting and mother's and children's stress increase the odds of having SB (OR > 2; *p* < 0.02) (43). Finally, one study determined that the prevalence of SB among individuals with tonsil hypertrophy, restricted tongue mobility, and nasal obstruction was 90.9% (*p* < 0.05) (44).

### Summary of studies based on EMG and PSG

One study evaluated the accuracy of PSG as a tool to assess SB in children (45), another determined the correlation between an EMG device (Grindcare measure) and PSG to evaluate SB/MMA in children (46), and two more evaluated the sleep architecture and physiology in children with SB (47, 48). One last investigation observed the effect of a Mandibular Advancement Device (MAD) to reduce SB by means of the BiteStrip® EMG device and obstructive sleep apnea by means of the ApneaLinkTM Plus in children (49) (Table 5). Subjects were recruited either among patients of a medical hospital (49) or university dental clinics (45–47). The assessment of SB with portable devices used in both studies with EMG for in-home recording were based on single-channel recording (46, 49). In one study, the researchers placed the EMG surface electrode (Grindcare measure) on the temporal muscle for five nights (46) and, in the other one, the BiteStrip® (49) was placed in the masseter muscle for 60 nights.

**Table 5:** PICO-like structured Reading of reviewed articles using EMG and/or PSG either as assessment strategy or outcome measure.

Study First author and year	Patient (Population)	Intervention (Design)	Comparison (Control Group)	Outcome	Conclusions	Quality SCORE
Restrepo, 2018 (46)	Parents of 12 7–11 year-old children reported “sometimes SB” and 19 reported “usually SB” in the Children's Sleep Habits Questionnaire (CSHQ).	Design: Cross-sectional. Measurements: A consecutive five = night electromyography (EMG) recording was performed with the GrindCare Measure (GCM). On the last night, children underwent a single-night PSG study together with the GCM. SB episodes in the PSG were interpreted according to cut-offs by Lavigne and the correlation between measurements with GCM and SB/PSG were determined.	Parents of 16 7–11-year-old children reported “rarely SB” in the CSHQ.	There were no significant correlations between GCM and PSG measurements.	EMG measurement with GCM was not accurate enough to detect the same PSG/SB activity in children. No advantage was found of multiple assessment for five nights with GCM.	85%^a^
Shiraishi, 2021 (47)	Fifteen 7–13-year-old children with MMA index ≥ 2 episodes per hour were considered to have SB and included in the patient group.	Design: Cross-sectional Measurements: A single-night PSG recording was performed. MMA was scored by according to adult cut-offs according to Lavigne et al. Sleep cycle was divided into nREM and REM sleep segments and the frequency of MMA, transient arousal and movement, and cortical and cardiac activities were then quantitatively analyzed in relation to sleep cycles.	Eighteen 7–13-year-old Children with MMA index <2 episodes/hour in the PSG were classified as controls.	There were no significant differences between sleep architecture and sleep stage of children with and without PSG/SB. In sleep cycles, SB children showed more frequent MMA in all sleep stages than children in the control group, while changes in cortical and autonomic activities were not different between children with and without SB. In SB children, MMA was frequent in nREM stage 3 and was associated with increases in cortical beta activity and arousal; more than 70% of MMA occurred with cortical and motor arousals.	MMA was associated with arousal and alteration in sleep cycles in children with SB.	78%^a^
Restrepo, 2023 (48)	Forty-three children aged 7–12 years (mean age: 9.4 ± 1.3) with SB (assessed with parental reports through the CSHQ and PSG with MMA index ≥ 4 episodes per hour).	Design: Cross-sectional Measurements: Children underwent a two-night polysomnographic (PSG) study in a sleep laboratory, for accommodation (first night) and data collection (second night). Data on sleep architecture (total sleep duration (TSD), sleep efficiency (SE), sleep onset latency (SOL), REM and nREM sleep duration and proportion and microarousals/hour during REM and nREM sleep) and episodes/hour of SB/MMA were recorded. Single and multiple-variable linear regression analyses were performed to assess the correlation between data on sleep architecture (predictors) and SB/MMA (dependent variable).	There was no a control group.	Shorter TSD, REM and nREM stage 1 sleep duration, longer SOL and more microarousals/hour during REM were found to be predictors of masticatory muscle activity related to SB in children (*R*^2^ = 0.511).	Masticatory muscle activity related to SB Is associated with altered sleep architecture in children [shorter total sleep duration (TSD), shorter nREM and REM sleep and higher microarousals during REM sleep]. Nevertheless, the clinical significance of these findings need to be demonstrated in future studies.	90%^a^
Restrepo, 2017 (45)	Parents of twenty-one 7–11 year-old children answered “Sometimes”, “Usually”, or “Always” to the question of the CSHQ “Has your child ground their teeth during the last week?”	Design: Cross-sectional Measurements: Parents of children filled out the CSHQ and a diary of STG during sleep over 5 days. On the fifth day, children underwent a one-night PSG in a sleep laboratory. PSG/SB was estimated by assessing MMA in masseter and temporalis EMG traces. EMG events separated by 3-s intervals were recognized as MMA/SB episodes, more than six bruxism bursts per episode, and/or ≥25 bruxism bursts per hour of sleep. When the first two criteria were satisfied, but ≤25 bruxism bursts per hour of sleep were present, at least two episodes with STG were required for a PSG/SB diagnosis. PSG data was compared with data of the CSHQ and the diary to determine correlation of parental-report of SB with PSG/SB.	Sixteen parents of 7–11-year-old children parents answered “Never” or “Rarely” to the question of the CSHQ “Has your child ground their teeth during the last week?”	The assessment with the CSHQ showed weak correlation and fair agreement (*r* = 0.34 and *k* = 0.40) with PSG/SB adult criteria in children. Multiple-observation evaluation of STG with the diary presented moderate correlation and agreement (*r* = 0.50 and *k* = 0.48) with PSG/SB data using adult criteria.	Parental reports of SB were not correlated with PSG/SB adult criteria in children.	75%^a^
Modesti-Vedolin, 2018 (49)	Eighteen 7–10-year-old patients were selected. Sleep bruxism was assessed with the BiteStrip®	Design: Quasi-experimental. Intervention and measurements: Sleep disorders were assessed with the Sleep Disturbance Scale for Children (SDSC). The clinical diagnosis of OSAS was confirmed with a type 3 portable monitor device (ApneaLinkTM Plus). A silicon-based material, MAD, was used by patients for 60 days, and the results were compared to baseline.	The same patients were evaluated before and after intervention.	There was a reduction from moderate to absence of SB in 12 out of 18 children.	MAD may be considered as an alternative to the OSAS treatment and useful for the reduction of SB in children.	56^b^

^a^ Quality assessment with STROBE.

^b^ Quality assessment with Qu-ATEBS.

All studies were based on a dichotomous assessment of presence/absence of bruxism, based on a cutoff threshold of two or four EMG episodes per hour of sleep (45–49), even though in one study, the number of EMG events were considered as a continuous variable (48).

As for the findings, two studies did not retrieve any significant associations between SB/MMA and parental-reported SB (45) or between EMG for in-home recording with the Grindcare Measure and SB/MMA in the PSG recordings (46). Regarding the behavior of sleep in children with SB, sleep architecture and sleep MMA in the different stages of sleep significantly differed between children with and without SB. In sleep cycles, SB children showed more frequent MMA in all sleep stages than controls, while cyclic changes in cortical and autonomic activities did not significantly differ between the two groups. In SB children, MMA was the most prevalent in nREM stage 3 and was associated with increases in cortical beta activity and arousal. More than 70% of MMA occurred with cortical and motor arousals (47). Regarding treatment, one study evaluated the effect of a mandibular advancement device (MAD) for the treatment of obstructive sleep apnea syndrome (OSAS) in pediatric patients and one of the outcome measures of that study was the reduction of SB, which went from moderate to absent in 12 of 18 patients (49).

## Discussion

The objective of this review was to compilate the best available evidence about SB in children from the last seven years and to give evidence-based recommendations for pediatric healthcare givers to treat bruxism in the clinic. The results showed that there is available literature using only parental-report of SB as well as parental-report and clinical symptoms of SB (e.g., tooth wear, Linea Alba, impressions in the mucosa and tongue, headaches, and orofacial pain on awakening, chewing and/or opening, and tenderness and pain on the temporal and masseter muscles during bilateral palpation) and SB/PSG. Epidemiological as well as case-control and intervention studies were found. The investigations dealt with genetics, sleep behaviors and architecture, lifestyle and habits, and quality of life, diet, and assessment of reliability of diagnostic tools and parental reports of SB.

The experimental studies addressed the intervention of SB from its origin, evaluating the effect of two types of orthopedic devices (e.g., McNamara and MAD) to reduce the obstruction of the airway aiming to reduce parental-reported SB and SB episodes. Below, each of the topics found in the review will be discussed.

### Genetic

According to the results of this review, there is an association between SB and expression of certain polymorphisms (41) of Dopamine and Serotonin, which are related to coping strategies, the regulation of the wake-sleep cycle and the regulation of hunger and satiety (50). Regarding the Dopaminergic genes, the DRD2, DRD3, and DRD5 receptors (51, 52) represent a predisposing genetic pattern for SB in adults, but DRD2 and catechol-o-methyltransferase (COMT) have been related to SB in children (42), as shown in this review. COMT and DRD2 are associated with disruption of the reward system in the brain (53), stress, authoritarian parenting patterns (54), and deleterious changes in the quality of life (55) of children. These patterns, in turn, have also been recognized in children with SB at the school stage (34, 36).

As will be mentioned later in this review, SB has a close relationship with OSA. In this regard, in adults, the DRD1 rs686 may indicate a predisposition to BS caused by obstructive sleep apnea and or hypopnea (OSA), and the HTR2A rs2770304 polymorphism could contribute to the association between SB and OSA (56). In this review of the literature, these polymorphisms were not related to SB in children. Further studies are necessary to determine the relationship between the genetic factors associated both with respiratory disturbances (e.g., OSA) and SB in children.

### Sleep architecture, GERD, and breathing disorders

Two studies were included in this review that deal with sleep architecture of SB in children (47, 48). One showed that SB was more frequent in nREM 3 (47), while the other indicated that sleep architecture of children with SB was more affected in REM than in nREM sleep (48). This is in agreement with the results of a previous investigation that found that the episodes of SB occur primarily during nREM 2 and during REM sleep (57). In the three studies (47, 48, 57), a relevant percentage of bruxism episodes were associated with arousal. Additionally, within the limitations of the study, Restrepo et al. (48) demonstrated that the odds of high masticatory muscle activity related to SB increased when there was high sleep onset latency, short sleep duration, short REM sleep duration, and high microarousals/hour during REM sleep.

A significant correlation between SB and snoring has been supported by several studies in children (35, 44). Although studies have suggested concomitant occurrence of SB in individuals with OSA, a narrow upper airway, rather than an obstruction of the upper airway, could be a factor contributing to the relationship between snoring and SB in children (58). OSA and its relationship to sleep bruxism is related to an arousal response that is often generated by hypoxemia and respiratory difficulty that triggers a serotoninergic neuronal reaction (56). At the end of an apneic event, frequent snoring, mumbling, gasping, and or STG occur (59). It is imperative to recognize these symptoms early, as children with SB can have a high likelihood of showing problematic daytime behavior that can also be frequently associated with sleep problems (60). Thus, evaluating the upper airway and using other diagnostic tools such as cephalograms and panoramic x-rays to determine airway dimensions and/or obstruction is mandatory in the dental room to search for a transdisciplinary assessment and intervention.

GERD acidifies the gastric tract and causes contraction of the airway, triggering SB/MMA in order to increase the buffer capacity by stimulating the salivary glands (61). Bruxism patients who experience GERD for extensive periods of time are prone to severe tooth wear. Gastric juice has greater erosive effects on both enamel and dentin (62) compared to acids from diet (63). This may be the reason why, when excluding from studies children with GERD, increasing frequency of SB was not correlated with the severity of tooth wear, as demonstrated in one of the articles included in this review (39). According to this evidence, it is mandatory to change the procedures to determine the origin of tooth wear and rethinking tooth wear as a diagnostic criterion to assess SB in children.

### Habits, diet, and lifestyle

Quality of life (QoL) is emerging as an important outcome measure in the current medical literature, but available data on its relationship with SB in children are still scarce. As stated before, polymorphisms in DRD2 related to bruxism phenotypes in children (42) is a genetic variation in the dopamine receptor D2 (DRD2) that may alter dopamine signaling and modify the rewarding effects of food (64) and videogame playing (65) and could explain the relationship between SB, sugar, and screen overuse (34). The consumption of added sugar and excessive screen-time is increasing worldwide (66), increasing the prevalence of sleep problems (67, 68), psychosocial disorders (69, 70), lack of cortisol homeostasis (71), depression and hostility, and Attention Deficit Hyperactivity Disorder-related symptoms (72). All these issues have also been associated with SB in children (35).

On the same hand, alterations in QoL have been associated with SB in children, particularly effects on working memory and emotional regulation (36). Excessive screen-time and added sugar consumption are also risk factors for the same QoL issues (73, 74).

### Stress, anxiety, and oral parafunctions

Anxiety in childhood is a frequent occurrence (75). However, it is underdiagnosed because of diverse symptomatology according to the different phases of development. Additionally, the concept of education is changing, and family structure and parenting style are also changing. Parenting requires spending a lot of time and resources on caring for and nurturing children (75). In addition, the unavailability of educational resources, the comparison between peers, and the lack of parenting experience increase parents' and children's tension and anxiety. Mothers have the highest influence on children, as they have the most contact with them (75). This may be the reason why studies have found that there is a correlation between mothers' and children's emotions. As a matter of fact, one of the studies included in this review (43) found that elevated anxiety levels of mothers increase the risk of having SB in children. Additionally, the same study found nail-biting as a predictor of SB and proposed that this could be a consequence of anxiety (43). This is the main reason why it is so important to evaluate the children's relationship with their mothers and the rest of their families when addressing SB in children in the clinic.

### Intervention of SB

The two investigations that were included in this review regarding intervention were focused on improving airway patency (40, 49) in order to reduce the mechanism that triggers SB in children. Other studies evaluating strategies to treat anxiety, psychological issues, and parafunctional habits associated with SB were not included in this review, even though they are extremely necessary.

The study by Giannasi et al. (40) evaluated the parental reports of SB before and after palatal expansion with the McNamara device to increase nasal volume and airflow. The other investigation (49) increased oropharynx dimensions by using MAD in children with respiratory disturbances and the measurement of SB was made with PSG (49). In both cases, the report of SB and SB/MMA decreased.

### Quality of the studies

The quality of studies about bruxism in children varies widely. All studies included in this systematic review were well-conducted, using appropriate study designs and methods. However, there are limitations, such as small sample sizes, lack of appropriate control groups and control of bias, and unclear measures of outcomes. Additionally, there is a lack of consensus about the assessment and treatment of bruxism in children, which leads to variability in study results.

One of the main challenges in conducting studies about SB in children is the difficulty in diagnosing the condition. Bruxism is often identified by parental reports of grinding or clenching teeth during sleep and/or clinical examination, but even the reports and clinical examination are not standardized or accurate in identifying the condition.

Another challenge is the lack of consensus about the etiology of bruxism. Some studies suggest that it is related to psychological factors, such as anxiety or stress, while others point to quality of life, lifestyle and habits, diet, or other medical conditions. This variability in etiology can make it challenging to design studies that adequately control for confounding factors and is difficult to review the studies to synthetize the evidence.

Despite these challenges, there have been several well-conducted studies on SB in children that provide insights into the prevalence, risk factors, outcomes, and intervention of the condition. These studies have highlighted the importance of paying attention to what is generating SB and intervening when necessary.

However, more high-quality studies are needed to better understand the underlying mechanisms, risk factors, and effective interventions for bruxism in children. Future studies should strive to use appropriate study designs and methods, include appropriate controls and measures, and use consistent definitions and diagnostic criteria to improve the quality and comparability of study findings.

## Conclusion

**Figure 2:**
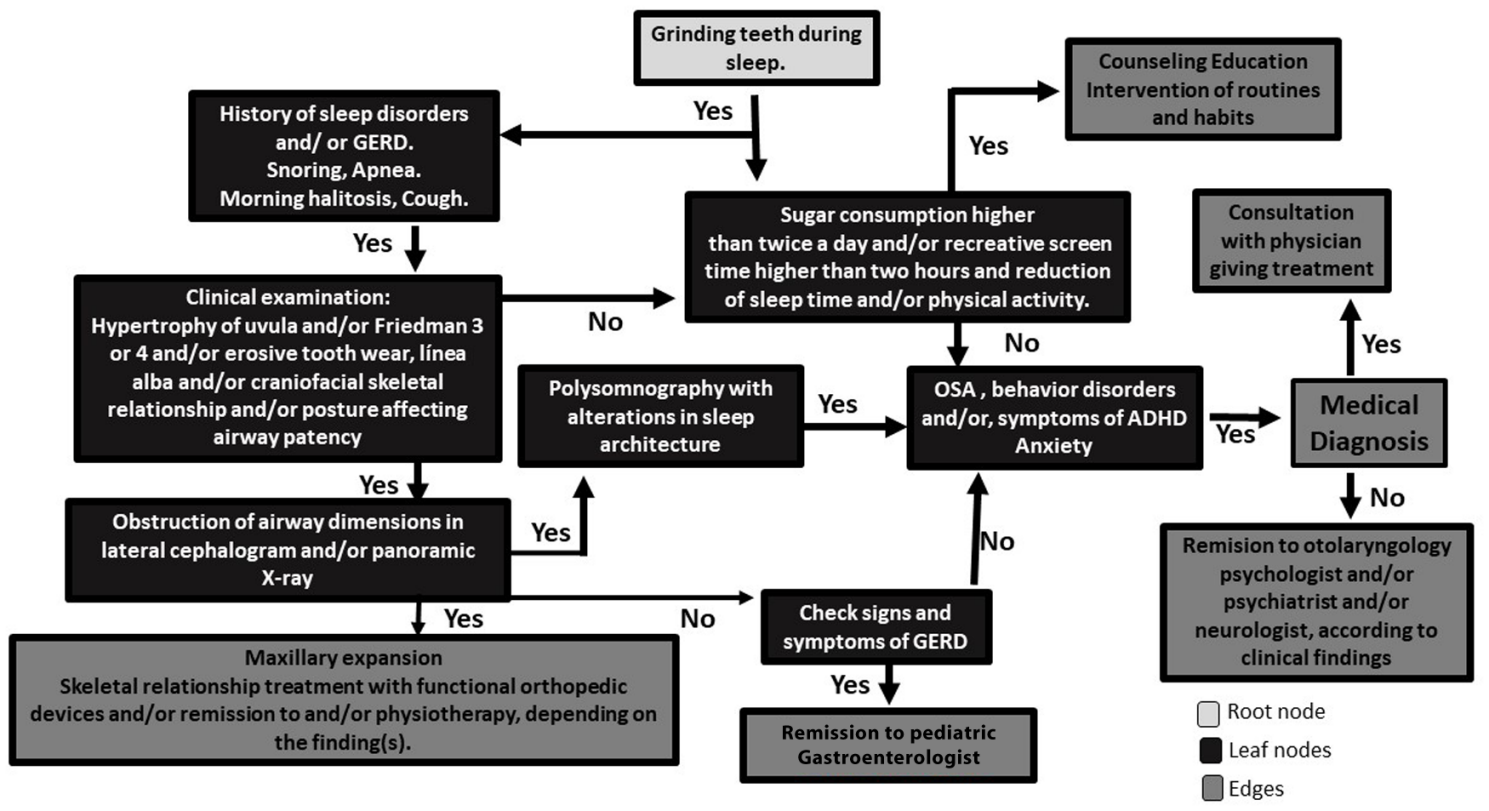
Flowchart based on an algorithm to assess and intervene against SB in the clinic. Starting from the root node (grinding teeth during sleep), go to the next nodes (search for symptoms of underlying diseases and dietary and lifestyle habits). Once you reach the leaf node, the node tells you the predicted outcome. Then the edges are subsets to look at, depending on the Yes/No answers to the steps of the algorithm.

Sleep bruxism is a symptom of underlying health, biopsychosocial, lifestyle, and diet conditions of children and their families. According to the findings in this review, methods of SB assessment are heterogeneous and hamper a reliable comparison of the results. However, with the available literature, it is possible to propose a flowchart based on an algorithm to assess and intervene with SB in the clinic (Figure 2). This algorithm contains the main variables that have been studied regarding SB in children and proposes a transdisciplinary approach.
